# The Use of ZrO_2_ Waste for the Electrolytic Production of Composite Ni–P–ZrO_2_ Powder

**DOI:** 10.3390/ma14216597

**Published:** 2021-11-02

**Authors:** Jolanta Niedbała, Magdalena Popczyk, Grzegorz Benke, Hubert Okła, Jadwiga Gabor, Roman Wrzalik, Arkadiusz Stanula, Andrzej S. Swinarew

**Affiliations:** 1 Łukasiewicz Research Network—Institute of Non-Ferrous Metals, Sowińskiego 5, 44-100 Gliwice, Poland; jolanta.niedbala@imn.lukasiewicz.gov.pl (J.N.); grzegorz.benke@imn.lukasiewicz.gov.pl (G.B.); 2 Faculty of Science and Technology, University of Silesia, 75 Pułku Piechoty 1A, 41-500 Chorzów, Poland; hubert.okla@us.edu.pl (H.O.); jadwiga.gabor@us.edu.pl (J.G.); roman.wrzalik@us.edu.pl (R.W.); 3 Institute of Sport Science, The Jerzy Kukuczka Academy of Physical Education, Mikołowska 72A, 40-065 Katowice, Poland; a.stanula@awf.katowice.pl

**Keywords:** Ni–P–ZrO_2_ powder, composite powder, chemical composition, surface morphology, energy-dispersive spectrometry (EDS), X-ray diffraction (XRD), element distributions maps

## Abstract

Ni–P–ZrO_2_ composite powder was obtained from a galvanic nickel bath with ZrO_2_ powder. Production was conducted under galvanostatic conditions. The Ni–P–ZrO_2_ composite powder was characterized by the presence of ZrO_2_ particles covered with electrolytical nanocrystalline Ni–P coating. The chemical composition (XRF method), phase structure (XRD method) and morphology (SEM) of Ni–P–ZrO_2_ and the distribution of elements in the powder were all investigated. Based on the analyses, it was found that the obtained powder contained about 50 weight % Zr and 40 weight % Ni. Phase structure analysis showed that the basic crystalline component of the tested powder is a mixed oxide of zirconium and yttrium Zr_0.92_Y_0.08_O_1.96_. In addition, the sample contains very large amounts of amorphous compounds (Ni–P). The mechanism to produce the composite powder particles is explained on the basis of Ni^2+^ ions adsorption process on the metal oxide particles. Current flow through the cell forces the movement of particles in the bath. Oxide grains with adsorbed nickel ions were transported to the cathode surface. Ni^2+^ ions were discharged. The oxide particles were covered with a Ni–P layer and the heavy composite grains of Ni–P–ZrO_2_ flowed down to the bottom of the cell.

## 1. Introduction

Nano-metal-ceramic powder composites are interesting materials due to their properties. Compared with composite microcrystalline powders, they are characterized by higher hardness. The reduction of the grains to nanometric sizes for electrochemically produced n-nickel increases the Vickers hardness from 140 to 650. It has also been observed that the nanocrystalline structure reduces the wear rate, increases corrosion resistance, and improves magnetic properties [1,2,3].

N-materials lose their properties at elevated temperatures due to grain growth. The experiments conducted for n-nickel revealed that grain size reduction did not increase the hardness. In this case, the opposite behavior of Hall–Petch was observed [4]. To overcome this, syntheses of ceramic composites with a metal matrix are carried out. It is essential in this case that the particles prevent or suppress the migration of the grain boundaries, thereby increasing the thermal stability of the material. The ceramic particles in the metal matrix should increase the hardness by limiting the shift of the grain boundaries and the dislocation movement. This depends on the number of ceramic particles and the uniformity of their distribution. For this reason, according to the Niihar concept, a nanocomposite with a metal matrix is a good material [5,6,7,8,9,10,11,12,13,14]. Nanocrystalline metals and alloys can be produced in many ways. The methods used are mainly ball milling, inert gas condensation and electrodeposition. The electrodeposition method requires the presence of dispersed ceramic particles in the electrolyte. The electrodeposition method enables the synthesis of many composites by combining electrolytes with various ceramic particles. Moreover, the process enables deposition rates in the range of 100 µm/h, is low-temperature, and does not require a vacuum. An additional advantage is the fact that you can quickly implement production on an industrial scale. Existing plating plants can still be used. The experiments conducted for nickel-based materials confirmed the high catalytic activity of hydrogen evolution and good corrosion resistance in aggressive environments [5,6,7,15,16,17]. The production of an electrode material with a highly developed, rough, or porous surface is possible by introducing composite components, alloying with other elements, or other modifications. Such operations are aimed at improving the properties of composite materials and increasing their catalytic activity [5,6,7,8,9,10,11,12,13,14,15,16,17,18,19,20,21,22,23,24]. Carbides, polymers, silicides, nitrides, and oxides are several types of particles used to improve the mechanical, physicochemical or electrocatalytic properties of composites [5,6,7,8,9,10,11,12,13,14,15,16,17,18,19,20,21,22,23,24].

Recent years have seen the development of electrodeposition technology, which consists of the introduction of powdered components into the metal matrix. This technique allows one to obtain new types of composite materials that can be used, for example, as electrode materials. The method is an electrolytic generation of materials by co-depositing particles dispersed in the electrolyte and producing a matrix + powder composite [15,16,17,23,24,25,26]. Among these composites, metal-containing materials such as Al, Ti, V, Mo, W are of particular importance. This is because these metals cannot be directly co-deposited in cationic form from aqueous solutions. Therefore, these metals are introduced in the form of powders during the co-deposition process [15,16,17,23,24,25,26,27,28,29,30].

Electrode materials with a highly developed, porous surface can also be produced by PVD or CVD methods. For economic reasons, a cheaper base (e.g., carbon steel) can be used in this case, a composite powder containing a ceramic material with a nanocrystalline coating is then applied to this substrate. The purpose of this work was to obtain crystalline composite powder with nano- or amorphous coating on crystalline particles.

## 2. Materials and Methods

**Figure 1:**
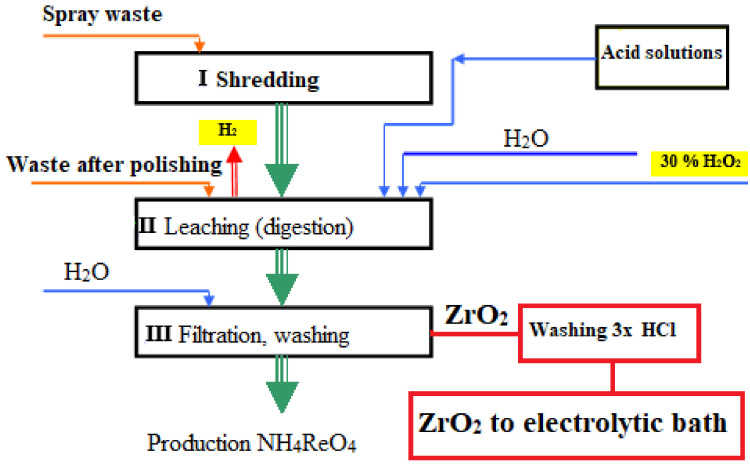
Diagram of zirconium oxide production used in the composite electro-fabrication process.

The Ni–P–ZrO_2_ powder was obtained from a galvanic bath contained the ceramic ZrO_2_ powder. The galvanic bath contained: 30 g/dm^3^ NiSO_4_, 48 g/dm^3^ NaH_2_PO_2_, 10 g/dm^3^ NH_4_Cl, 8 g/dm^3^ H_3_BO_3_, 20g/dm^3^ CH_3_COONa. Reagents of analytical purity (Chempur Poland) and deionized water were used for the solution. To this bath, an appropriate amount of ZrO_2_ powder was added (10 g/dm^3^). The zirconium oxide used in this study is obtained in the process of pulping waste from the plasma application of protective coatings from superalloys and zirconia oxide on metal surfaces. During sputtering, a part of the material settles on the walls of the spraying chamber or is stopped by the dust removal system. The recovered material is recycled to a small extent. And due to the lack of processing methods, it is stored. Therefore, the method of digesting these wastes was developed and patented [31]. After reconstitution and filtration, the resulting solution can be directed to the recovery process by known methods for rhenium and other metals. ZrO_2_ remains in the form of a raw, polluted precipitate. For purification, the raw oxide was washed three times in concentrated HCl. As a result, the obtained oxide contained the main additives Y and Hf. The content of other impurities was minimal (Figure 1).

The electrodeposition process was carried out in an electrolytic cell (V = 600 cm^3^, ϕ = 8 cm), the volume of the nickel bath was 500 cm^3^, and the solution was mechanically stirred (300 rpm) to keep the ceramic powder (ZrO_2_) in suspension. The temperature of the bath during deposition was 328–333 K. The electrodes (anode and cathode) were placed parallel to the walls and vertically in the vessel. The composite powder was fabricated using a copper cathode and nickel anode (Ni 201). The geometric area of the cathode was 4 cm^2^, and the anode 8 cm^2^. The copper plates were prepared by mechanical polishing and chemical activation in an alkaline bath (170 g/dm^3^ NaOH), and hydrochloric acid 1:1. The deposition process was performed under galvanostatic conditions in the current density 100 mA/cm^2^ for 4 h. During the electrolytic process, the powder composite settled on the cathode and then fell to the bottom of the vessel over time. Because the mixing was carried out continuously, the obtained composite grains were entrained in the suspension of oxide in the bath. After completion of the process, the obtained material was filtrated. The obtained composite Ni–P–ZrO_2_ powder was washed three times with warm distilled water (323 K), and in the next step was dried in a laboratory dryer at a temperature of 70 °C for 5 h. The chemical composition of the Ni–P–ZrO_2_ composite powder was determined by X-ray fluorescence spectroscopy using a WD XRF spectrometer model ZSX Primus by Rigaku (Osaka, Japan). The source of the radiation was a lamp operating at 50 kV and intensity of 50 mA. LiF200 crystal was used to measure the fluorescent radiation characteristic of the elements from potassium to uranium (according to the position in the periodic table). For the fluorescence measurements of phosphorus a germanium crystal was used. Each maximum recorded in the spectra of the fluorescence intensity from the 2 Θ angle, called the peak or line, was compared with the theoretical angles of 2 Θ tables for the range of analyzed elements. In the case of the theoretical consistency of this angle with the angle assigned to the line recorded in the spectrum, such a line was marked as a line characteristic for a given element. Oxygen content in the Ni–P–ZrO_2_ powder was determined by the IR method. Structural investigations were conducted with an XRD 7 X-ray diffractometer from Seifert-FPM (Ahrensburg, Germany). Characteristic radiation Co K_α_ and Fe filters were used. The analysis was carried out in the range of 2 Θ angles from 10° to 100°, which corresponds to the range of interplanar distance d_hkl_ from 1.027 nm to 0.1168 nm. The step method was used, where the length of the measurement step was 0.04° 2 Θ and the count time at the measurement point of 2 s. The identification of crystalline phases in the sample was made on the basis of Seifert software and PDFD 2 catalog data from 2007 by ICDD. Chemical analysis of all obtained coatings was performed by ZSX Primus X-ray fluorescence spectrometer with wavelength dispersion (Rigaku, Tokyo, Japan). The spectrometer is equipped with a 4-kW lamp with a rhodium anode, a set of filters, and crystals, allowing the analysis of elements in the range from boron to uranium. Under the influence of the lamp radiation, the atoms in the sample emit their own characteristic radiation, which, after passing through the optics system, reaches the detector. Measurements of samples were made using the so-called non-standard-semiquantitative X-ray fluorescence spectrometry (XRF) method with the use of a mask allowing the measurement of a surface with a diameter of 1 cm and with switched off the sample rotation.

The research was carried out using X-ray microanalysis (EPMA) using the JEOL X-ray microscope JXA 8230 (Tokyo, Japan). Measurements were made at 15 kV acceleration and 30 nA beam current. The qualitative and quantitative composition in selected places of the sample was determined using the energy dispersive spectroscopy (EDS) method. Quantitative analyzes of the chemical composition were carried out using the standards of all analyzed elements. Element distribution maps were made using the wavelength dispersion (WDS) method with a much better resolution compared with the EDS method. Powder morphology shows images in the light of secondary electrons (SEI).

Three dimensional visualization of the samples was performed using computer microtomography (v|tome|x, GE Sensing & Inspection Technologies, Phoenix | x-ray, Wunstorf, Germany) using the parameters from Table 1. The determined parameters allowed for the registration of an image with optimal contrast and resolution.

The topography of the samples was imaged using an atomic force microscope (AFM), NanoScope E (Digital Instruments, Santa Barbara, CA, USA) equipped with an NP-S scanning probe (NanoProbeTM, Veeco, Plainview, NY, USA). Measurements were carried out in contact mode. The nominal spring rate of the V-shaped cantilever used was 0.32 N/m and the constant force applied was about 20 nN. Measurements were performed with the use of the AS-12 ″E″ scanner, thanks to which the maximum size of the images in the horizontal directions X and Y was 13 μm and the maximum height was 3.8 μm. The images were acquired with a transverse resolution of 10 nm and a height resolution of 1 nm. The obtained images were analyzed with the WSxM software package.

## 3. Results and Discussion

In the initial stage, the analysis of zirconium oxide, formed as sludge during the leaching of waste from the plasma treatment of metal surfaces, was performed. The composition of the zirconia used was as follows (weight %): 68–73% Zr; 2–5% Y; 1–1.5% Hf; 0.05% Ca; 0.05–0.08% Fe; 0.05–0.1% Cr; 0.1–0.3% Si; 0.5–0.8% Al; 0.05% Ni; 0.08% Co. In this material the basis was ZrO_2_, the main additives were the stabilizing oxides Y and Hf. However, the content of the rest of the components depended on the effectiveness of the leaching and was minimal. This defined oxide was introduced to a galvanic Ni–P bath. When the current flowed through the solution with the oxide suspension, nickel ions were adsorbed on the oxide particles. 

During the electrodeposition process, the adsorption of nickel ions takes place on the surface of the solid particles dispersed in the electroplating bath. An amorphous nickel coating is formed when the ions originally adsorbed on the particle surface are reduced at the cathode. Adsorption of Ni cations on the surface of the powder dispersed in the electrolyte facilitates their reduction. In this way, solid particles are incorporated into the electrolytically deposited matrix, or a metallic coating is deposited on the solid particles.

A similar phenomenon has previously been described with regard to oxides, metal powders or polymers (e.g., [10,11,14,19,22]). The novelty of this process was the use of nickel anode, which guaranteed a constant level of nickel ions in the galvanic bath. The production mechanism of composite powder particles is explained on the basis of the Ni^2+^ ions adsorption process on the metal oxide particles. Current flow through the cell forces the movement of particles in the bath. Oxide grains with adsorbed nickel ions were transported to the cathode surface. Ni^2+^ ions were discharged. The oxide particles were covered with a Ni–P layer, and the heavy composite grains Ni–P–ZrO_2_ flowed down to the bottom of the cell.

**Figure 2:**
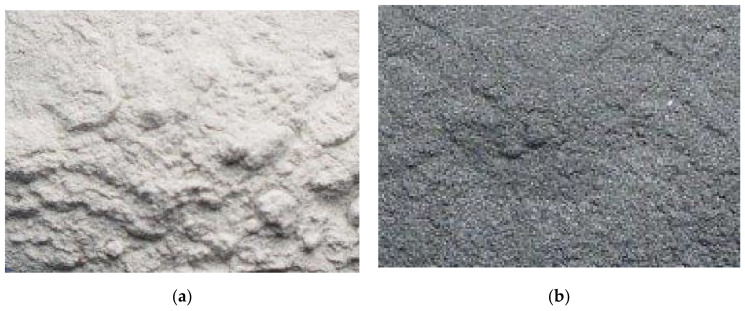
ZrO_2_ obtained as a sludge during leaching of waste from the plasma treatment of metal surfaces (**a**) and Ni–P–ZrO_2_ composite powder after the electrodeposition process (**b**).

Figure 2 shows the ZrO_2_ and Ni–P–ZrO_2_ powder. Photos were taken with a digital camera C-4000 ZOOM Olympus (Olympus America Inc., New York, USA). The graphite color obtained in Ni–P–ZrO_2_ is evidence of the presence of a metallic coating on the zirconia particles. The obtained composite material is finely powdered. In normal conditions, without protection, it does not tend to crack and agglomerate.

**Figure 3:**
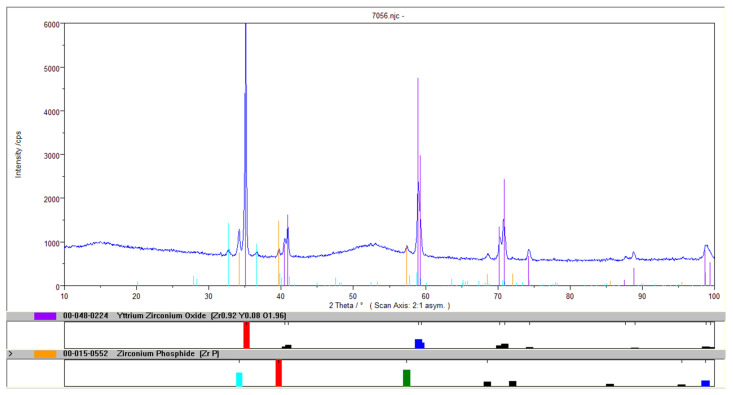
Diffractogram of the Ni–P–ZrO_2_ composite powder.

XRD analysis showed that the basic crystalline component of the tested powder is a mixed oxide of zirconium and yttrium Zr_0.92_Y_0.08_O_1.96_. The presence of small amounts of a few percent, ZrP zirconia and ZrO_2_ baddeleyite was also found. In addition, the sample contains very large amounts of amorphous compounds (on the order of several dozen percent), as indicated by the characteristic “wavy” shape of the graph’s background. The maximum of this amorphic signal occurs within the angular interval 50–55 Θ. The increased background and the angular lengths at which the signal appears allow the conclusion that it is derived from amorphous nickel. The shift of the signal is probably due to the presence of amorphous zirconium compounds found in the composite powder. Two variants of the sample diffractogram are included: with a full legend and with an exposed graph and a legend containing two crystalline compounds found in the largest quantities (Figure 3).

**Figure 4:**
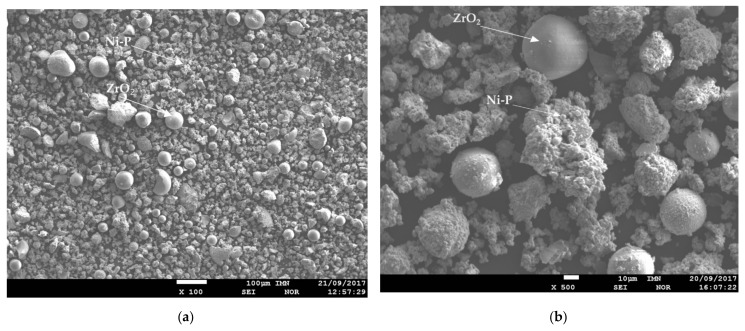

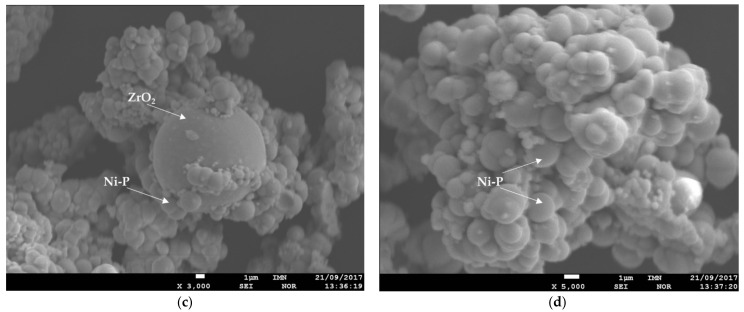
SEM images of surface morphology of the Ni–P–ZrO_2_ composite powder, magnification: (**a**) 100×, (**b**) 500×, (**c**) 3000× and (**d**) 5000×.

The surface morphology Ni–P–ZrO_2_ showed that composite powder is a mixture of different types of grains (Figure 4). On the SEM microscopic image, one can observe grains of zirconium oxide coated with Ni–P amorphous alloy, grains without an alloy coating, clusters of Ni–P alloys and other inclusions, e.g., gadolinium. Figure 4c shows the grain of zirconium oxide during the formation of the amorphous nickel coating, and next Figure 4d (at higher magnification) shows the same grain completely covered with nickel coating.

**Figure 5:**
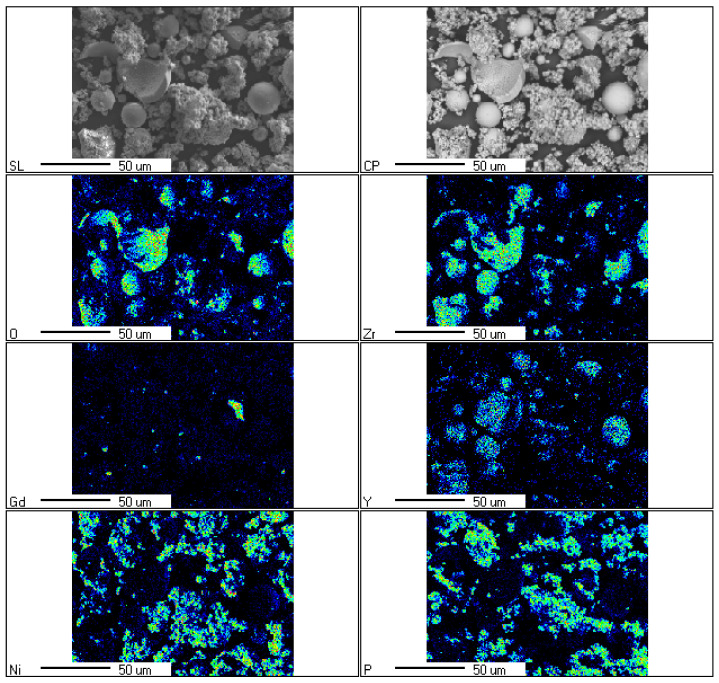
Element distribution maps, magnification 750× (area 160 μm × 120 μm).

Figure 5 presents the qualitative composition in selected place of the sample, determined using the energy dispersion (EDS) method with magnification 750 times, analyzed area 160 µm × 120 µm. The figure shows also the defective, broken grains of zirconium oxide. The presence of broken grains was the result of material recovered after the sputtering process in the pressure chambers. The analysis showed mainly Ni, Zr, P, and O. Y and Gd were also registered. Their presence resulted from the fact that they are the stabilizing factors of zirconium oxide. The presence of these broken grains is likely due to the fact that it is the oxide recovered from the waste after sputtering in pressure chambers.

**Figure 6:**
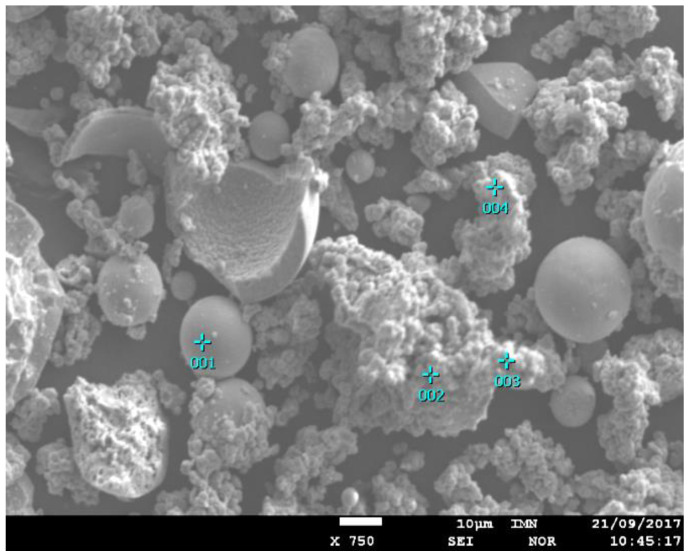
The analyzed area with selected analysis points and 1–4-point analysis.

Quantitative analyses of the chemical composition were carried out using the standards of all analyzed elements. A point analysis was performed by the EDS method (Figure 6, Table 2) and an analysis of the average content of elements in the sample using the XRF method (Table 3). The results of the point analysis are presented. They confirm the observations made on the basis of the SEM imaging.

The chemical composition analysis of the Ni–P–ZrO_2_ composite powder showed that the material contains almost (weight %) 50% Zr, 40% Ni, about 3.5% Y and P and 2% Gd (Table 3). The material also showed the presence of Si and Mn in an amount below 0.1%. 

When comparing the results of the point analysis with the average results from the entire volume of the sample obtained by X-ray fluorescence, it should be stated that the ZrO_2_ grains were in different degrees covered with amorphous nickel. SEM analysis showed that the degree of coverage does not depend on the size and degree of grain defect. In order to standardize the obtained material, further tests will be carried out. 

**Figure 7:**
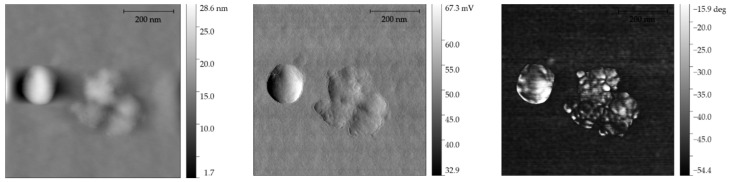
AFM images: height, signal error and phase signal. Visible zirconium oxide grain and coating material.

**Figure 8:**
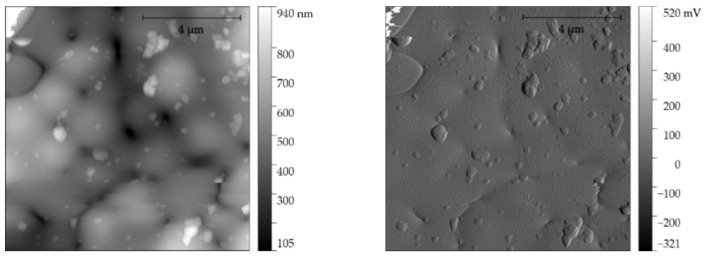
AFM image of height (topography) and signal error of large grain with overlap (captured at grain ripple).

The imaging in the atomic force microscopy technique revealed and confirmed the structure of the obtained particles as well as their size and static structure (Figure 7 and Figure 8). Performing this imaging confirms the data obtained with the use of computed tomography and also indicates the absence of low-energy agglomerations of particles. Single particles have a smooth surface structure and imaging of the structure was visualized with a very good signal-to-noise ratio; no galvanic defects were visualized.

**Figure 9:**
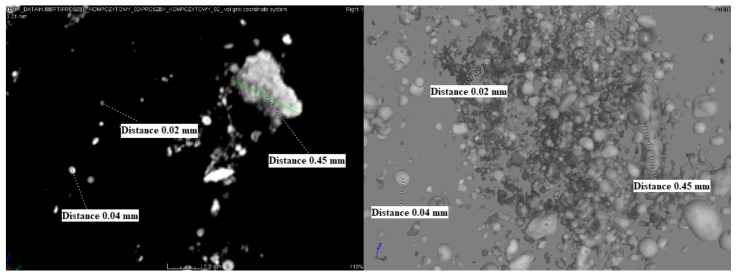

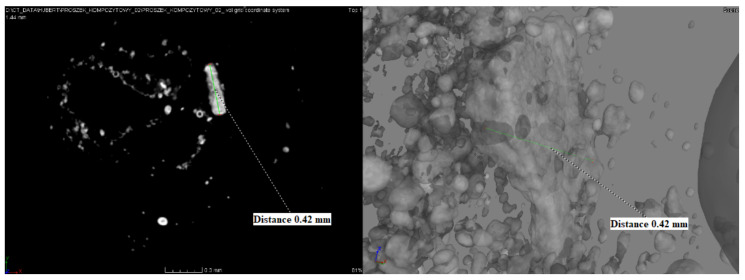
µCT registered morphology of the Ni–P–ZrO_2_ composite powder.

The recorded images in Figure 9 show the size and spatial distribution of the grains. The recorded images clearly show the heterogeneity of both the size and the steric system of the observed structures. This phenomenon may result from the decomposition and random deposition of zirconium oxide on the resulting crystal structures (nuclei), and the lack of agglomeration is clearly visible. 

The obtained Ni–P–ZrO_2_ composite material obtained by the electrolytic deposition method was thoroughly characterized. A point analysis (atom%) was performed using the XRF method, while the chemical composition (weight%) was described using the EDS method. Additionally, a composite phase composition analysis (XRD) was performed. Characterization of the surface morphology (SEM) was also carried out, documenting the presence of zirconium oxide particles coated with amorphous nickel in the obtained composite powder, which was additionally confirmed and visualized by the results of microtomographic and microscopic AFM analysis.

## 4. Conclusions

The proposed method allows the use of zirconium oxide recovered from waste generated at the pressure application of ZrO_2_ coatings. A composite material, zirconium oxide with Ni–P amorphous coating, can be obtained in the electrochemical process. The XRD analysis showed that the basic crystalline component of the tested powder was a mixed oxide of zirconium and yttrium Zr_0.92_Y_0.08_O_1.96_. The presence of small amounts of a few percent of the ZrP zirconia and ZrO_2_ baddeleyite was also found. The sample contains very large amounts of amorphous compounds (probably on the order of several dozen percent). The signal appears at angular lengths characteristic of amorphous nickel. The signal shift is probably caused by the presence of amorphous zirconium compounds found in the complex powder. The chemical composition of the Ni–P–ZrO_2_ composite powder showed that the material contains almost (weight %) 50% Zr, 40% Ni, about 3.5% Y and P and 2% Gd, and the content of other analyzed components was lower than 0.1%.

On the basis of µCT imaging, it can be concluded that the method used for composite production is properly selected because it does not cause agglomeration.

Subsequent studies will verify the method of preparation of the recovered ZrO_2_ powder, preliminary grinding, and differentiation of the conditions used during the electrolytic process for applying an amorphous alloy coating (mixing, time, current conditions). 

The results of the research show that the result of the experiment is a composite material in which, as assumed, nanocrystalline nickel was deposited on the ceramic zirconium oxide particles. The method used allows for the obtaining of a powder of this interesting material. A limitation is that the powdered composite does not allow it to be used as an electrode material, as it requires further processing, such as thermal treatment or pressing. This will allow researchers to take full advantage of its properties as an electrode material.

## Figures and Tables

**Table 1:** Parameters of microtomographic scans and used software.

**Voltage**	100 kV
**Current**	150 μA
**Number of images**	1500
**Image width**	2024 pixel
**Image height**	2024 pixel
**Timing**	333 ms
**Averanging**	5
**Skip frames**	1
**Voxel size**	2.5 µm
**Grayscale**	8-bit
**Detector type**	dxr 250
**Acquisition software**	Datos 2.0 (GE Sensing & Inspection Technologies, Phoenix|x-ray, Wunstorf, Germany)
**3D reconstruction software**	VGStudio MAX 2.1 (Volume Graphics, GmbH., Heidelberg, Germany)

**Table 2:** Results of the point analysis performed by the EDS method (atom %).

Point	Ni (%)	Zr (%)	P (%)	O (%)	Gd (%)	Y (%)
001	1.29	68.46	-	22.71	-	7.54
002	80.30	4.29	7.23	2.32	5.85	-
003	85.15	2.43	12.10	0.32	-	-
004	4.73	24.83	0.41	8.78	60.61	0.63

**Table 3:** Chemical composition of the Ni–P–ZrO_2_ composite powder.

Element	Ni	Zr	P	O	Gd	Y
**Content (weight %)**	39.8	49.1	3.56	0.69	2.02	3.57

## Data Availability

The data presented in this study are contained within this article.
